# Relationships of lower extremity and trunk asymmetries in elite soccer players

**DOI:** 10.3389/fphys.2024.1343090

**Published:** 2024-02-02

**Authors:** Tomas Maly, Mikulas Hank, Ferdia Fallon Verbruggen, Christian Clarup, Kirk Phillips, Frantisek Zahalka, Lucia Mala, Kevin R. Ford

**Affiliations:** ^1^ Sport Research Center, Faculty of Physical Education and Sport, Charles University, Prague, Czechia; ^2^ Department of Performance, AC Sparta Praha, Prague, Czechia; ^3^ Department of Physical Therapy, High Point University, High Point, NC, United States

**Keywords:** strength, power, performance, football, injury prevention, isokinetic, isometric

## Abstract

In light of previous research highlighting the prevalence of asymmetries in soccer players and possible links to injury risks, there is a crucial gap in the biomechanical understanding of complex relationships between lower extremity and trunk asymmetries in elite soccer players. The purpose of this study was to investigate the level, relationships, and differences among twelve different parameters of strength, morphological, and neuromuscular asymmetries in elite soccer players.

**Methods:** Elite male soccer players (*n* = 25, age 21.7 ± 3.9 years) were tested in the following tests: bilateral fluid distribution, hip flexor range of motion, postural stability, isokinetic strength of knee extensors and flexors, isometric lateral trunk rotation strength, eccentric strength of knee flexors, isometric bilateral strength of hip adductors, and vertical ground reaction force in counter-movement jump-free arms, counter-movement jump, squat jump, and drop jump tests. One-way ANOVA, Pearson’s coefficient (r), and partial eta squared (*η*_p_^2^) were used for data analysis.

**Results:** Significant differences in asymmetries were found in elite soccer players (F_11,299_ = 11.01, *p* < .01). The magnitude of asymmetry over 10% was in postural stability and drop jump parameters. The lowest magnitudes of asymmetries were in the fluid distribution of the lower limbs and the vertical ground reaction force during the take-off phase in squat jumps. The highest asymmetries between the dominant and non-dominant sides were found in postural stability and drop jump. A total of eleven significant correlations (*p* < 0.05, r = 0.41–0.63, R^2^ = 0.17–0.40) were detected between the analyzed asymmetries in elite soccer players. The lateral trunk rotation asymmetries were significantly correlated to vertical ground reaction force asymmetries and knee extensors.

**Conclusion:** Long-term exposure in elite soccer leads to unilateral biomechanical loading that induces abnormal strength and morphological adaptations in favor of the dominant side while linking lower limb and trunk strength asymmetries. By unraveling these complex relationships, we strive to contribute novel methods that could inform targeted training regimens and injury prevention strategies in the elite soccer community. The data should encourage future researchers and coaches to monitor and develop trunk strength linked to lower body kinematics.

## 1 Introduction

In elite soccer, the interaction between lower limb and trunk strength is crucial for the intricate demands of kicking (Carvalho et al., 2021), running, and rapid changes in movement direction (Hodges and Richardson, 1997; Hedrick, 2000; Liebenson, 2010; Nagahara et al., 2018; Niewiadomy et al., 2021). Santana (2003) mentioned that the diagonal pre-stretch in the trunk’s ventral musculature, also known as the “serape effect,” optimizes force production by providing the core muscles with an optimal length-tension environment. This mechanism is an excellent way to produce force between the shoulder and the opposite hip (Santana, 2003). The majority of soccer players have a preferential or dominant lower limb to carry out repetitive unilateral movements, such as kicking a ball or changing directions (Carey et al., 2001). When unilateral load is involved in sport-specific movement, the limb becomes preferred by neural-motor patterns, resulting in different morphological and strength asymmetries (SAs) (Maly et al., 2019). Studies suggest that genetic factors, such as the LRRTM1 and protocadherin11X/Y gene pair, not only play a role in handedness but also acknowledge the influence of accidental variation, developmental instability, and early fetal development (Annett, 1978; Yeo and Gangestad, 1993; Francks et al., 2007; Crow et al., 2009). Asymmetries may result in significant changes in the myodynamic characteristics of the muscle, particularly in the dominant leg (Fousekis et al., 2010a). According to Iga et al. (2009), soccer players rarely use both limbs with the same emphasis, and this preference is related to hemispheric brain dominance. As the specific adaptation to imposed demands (SAID) principle dictates, this creates a functional asymmetry (FA) as the limbs respond to their respective roles of force generation during movement (Barbieri et al., 2015), balance (Gstottner et al., 2009; King and Wang, 2017), and morphology (Mala et al., 2023). These functional asymmetries between limbs (BLAs) fluctuate due to the demands of the environment (Maloney, 2019). They can manifest in aspects ranging from gait mechanics to the change of direction differences (Nicholson et al., 2022). However, the largest BLA in soccer has been reported in lower limb strength (Nicholson et al., 2022), with this being affected by long-standing participation in soccer (Fousekis et al., 2010b; Maloney, 2019; Maly et al., 2021). Côté et al. (2007) reported that elite players achieved more hours in specific football training during childhood and adolescence compared to those who did not achieve professional status. Long-term and highly specific physical activity (especially with more frequent use of the dominant lower limb compared to the non-dominant) may give athletes an incentive to reproduce functional and morphological asymmetries (Fousekis et al., 2010c). Hanimann et al. (2023) compared the asymmetry in knee valgus (medial knee displacement during drop jump) and core stability (displacement during dead bug bridging exercise) in high-level soccer and alpine skiers athletes. The study reported a similar magnitude of asymmetries in athletes, but the effect on the direction of laterality was different, which means differences in sport-specific demands. The most predominately measured BLA has been knee extensor and flexor muscles (Fousekis et al., 2010a; Menzel et al., 2013; Nicholson et al., 2022) and ground reaction force or heights attained during jump testing (Menzel et al., 2013; Arundale et al., 2020; Read et al., 2021). Asymmetry evaluation in elite soccer has also expanded to eccentric hamstring strength (Bishop et al., 2022a), trunk strength (Kubo et al., 2010), hip strength (Ocarino et al., 2021), and hip range of motion (Ocarino et al., 2021). The reason for this expansive search for SA in soccer players is due to its link to performance deficit (Read et al., 2021), decreased soccer-specific skills (Maly et al., 2018), or increased injury risk (Mala et al., 2020; Markovic et al., 2020; Helme et al., 2021). However, no consensus exists on these effects (Bishop et al., 2022a). This may be because asymmetries are usually analyzed in isolation despite BLA adaptations occurring throughout the lower limbs of elite soccer players in response to long-term sports participation (Fousekis et al., 2010b; Maly et al., 2021). Given the significant role that the trunk plays in energy transfer in soccer, revealing the mechanical strategies and kinematics between the lower limbs, hips, and trunk during kicking and running, the available data on trunk rotational strength in elite soccer players and its correlation with lower limb strength is incomplete. In particular, the effects of unilateral loading and asymmetry on optimal trunk performance require further investigation. However, addressing these gaps in knowledge will be critical to improving athletic performance and reducing the risk of injury. Thus, a novel approach to this problem would be to analyze the relationship between different morphological, neuromuscular, strength, and power asymmetries of lower limbs and trunk muscles. The purpose of the study was to investigate the level, relationships, and differences between twelve different parameters of strength, morphological, and neuromuscular asymmetries in elite soccer players.

## 2 Materials and methods

### 2.1 Study design

A cross-sectional design was used in this investigation. The study overview was explained before testing, and signed informed consent was collected from all players. The study was approved by the Ethical Committee of the Faculty of Physical Education and Sport, Charles University, under approval no. 107/2021. The ethical document preparation and measurement taking were completed in accordance with the ethical standards of the Declaration of Helsinki and the ethical standards in sport and exercise science research (Harriss et al., 2017).

### 2.2 Participants

Elite male soccer players from the highest division of the Czech Republic (*n* = 25, age 21.7 ± 3.9 years) volunteered to participate in the study. The majority of the players were recent members of their national teams. The playing positions of the players were as follows: goalkeepers (*n* = 4), fullbacks/wingbacks (*n* = 5), central backs (*n* = 4), midfielders (n = 5), wingers (n = 4), and attackers (n = 3). In total, 19 players were classified as right-footed and 6 players as left-footed. The average years of football training experience of the players was 15.4 ± 3.9 years. The typical weekly training/match frequency in-season is shown in Table 1. Inclusion criteria included the following: 90% of training and competition availability for the last 2 months prior to measurement; free from any musculoskeletal injuries or medical conditions that may significantly affect physical performance. Exclusion criteria included the following: high resistance or strenuous training performance within the last 48 h that may affect the maximal physical performance and strength asymmetry manifestation; recent history of lower limb or trunk injury within the last 6 months to minimize the potential for injury-related strength asymmetries; and any knee surgery in their entire playing career that may cause increased strength asymmetry.

**TABLE 1:** Brief overview of weekly training/match in-season period in relation to a match day.

Day	Field-based training	Gym training	Match (min)
MD + 1	Recovery^a^, top-up (60 min)^b^	30 min^a^ ^,^ ^b^	
MD - 2	60 min		
MD - 1	60 min		
MD		20 min^c^	90
MD + 1	Recovery^a^, top-up (60 min)^b^	30 min^a^ ^,^ ^b^	
MD - 1	60 min		
MD		20 min^c^	90

MD, match day.

^a^ Recovery and gym session for players who played for more than 45 min.

^b^ Field-based session includes players who either did not appear in line-up or played less than 45 min.

^c^ Prime pre-match session with the aim to use the post-activation potentiation effect.

### 2.3 Data collection

Measurements were taken before the beginning of the regular season 2022/2023 in the morning from 9:00 to 11:30 a.m. The players were not exposed to any exhausting physical load 2 days before testing.

#### 2.3.1 Anthropometric data

Body height was measured using a digital stadiometer (SECA 242, Hamburg, Germany), and body mass was measured using a digital scale (SECA 769, Hamburg, Germany).

#### 2.3.2 Morphological asymmetries

The bilateral fluid distribution in the lower limbs (MA_FD) was assessed using a multi-frequency bio-impedance analyzer (MC-980MA; Tanita Corporation, Tokyo, Japan). MA_FD was calculated as a percentage difference between the dominant vs non-dominant limb. Dominancy was assessed by determining which limb was preferred by the participant to kick a ball (Maly et al., 2019). To ensure consistency for bio-impedance measurements (Kyle et al., 2004), the procedure was conducted in the morning from 9:00 to 11:30 when the participants had not been exposed to various foods and hydration during lunchtime. This approach accounts for the potential limitations of three-frequency analyzers, such as the presence of material in the human body and hydration level, which may influence the measurements (Cridlig, 2013).

#### 2.3.3 Range of motion

Range of motion in hip flexion (HIPS_ROM) was performed as previously described by Cameron and Bohanon (1993). It was an active straight leg raise test. The examiner fixed the contralateral leg in place while the player raised their leg as far as possible. HIPS_ROM was measured by using a VALD DYNAMO goniometer (Vald Performance, Queensland, Australia), which was positioned and fixated on the outer femur.

#### 2.3.4 Neuromuscular asymmetry

Neuromuscular asymmetry was tested by the multi-sensory FOOTSCAN platform (RS scan; Belgium; 0.5 m × 0.4 m; approximately 4,100 sensors; sensitivity from 0.1 of N.cm^-2^; sampling frequency 500 Hz) during posturographic examination. Postural stability (PS_COP) was tested by single leg stance (flamingo stance). The total distance of the center of pressure excursion was recorded for 60 s for each leg, while the non-standing leg was in a semi-flexed knee position, as previously described (Marencakova et al., 2018).

#### 2.3.5 Isokinetic strength of knee extensors and flexors

The isokinetic peak torque of knee extensors and flexors was measured in concentric muscle contraction at 60°·s^-1^ using the Cybex Humac Norm isokinetic dynamometer (Cybex NORM ^®^, Humac, CA, United States). There was high reliability of peak muscle torque in isokinetic testing on Cybex Humac Norm for knee extensors (ICC = 0.98, 95% CI = 0.95–0.99) and flexors (ICC = 0.95, 95% CI = 0.88–0.98) (Impellizzeri et al., 2008). The testing protocol consisted of three attempts, with the maximal effort during knee flexion and extension. The bilateral ratio of knee extensors (ISOK_QQ) and flexors (ISOK_HH) was expressed as the percentage differences of peak torques between the legs. The torque was gravity-corrected, and dynamometer calibration was performed in accordance with the manufacturer’s instructions. After five submaximal warm-up repetitions, the participants performed three repetitions with maximum effort. Visual feedback and verbal stimulation were provided during the testing.

#### 2.3.6 Isometric lateral trunk rotation strength

**FIGURE 1:**
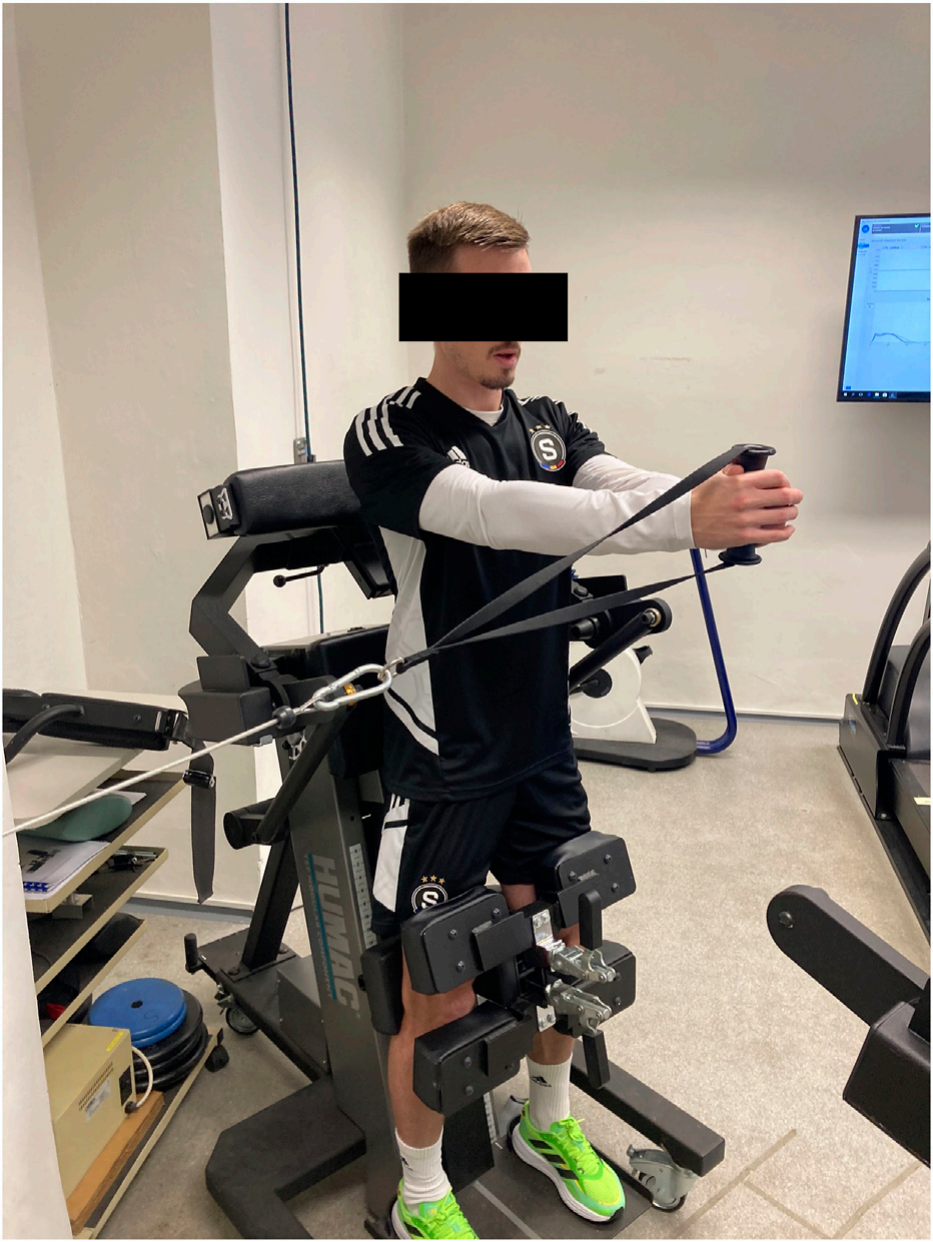
Maximal voluntary contraction test of trunk rotational strength using the isometric mode of the Humac NORM dynamometer with trunk modular and wheel components (CSMi, Stoughton, MA, United States).

The isokinetic dynamometer device was also used for the isometric lateral trunk rotation strength (ISO_TRUNK) test. The maximal voluntary contraction (MVC) of ISO_TRUNK measures was obtained with an additional Trunk Modular Component (CSMi, Stoughton, MA, United States) and the Humac NORM wheel attachment (CSMi, Stoughton, MA, United States). The peak force of MVC was represented in kilograms (kg) by the manufacturer and, consequently, in the percentage of individual body weight (%BW), which was used for further analysis. Before testing, a standardized warm-up of three sets of 10-s planks, three sets of six repetitions (each side) of the bird–dog exercise, and three sets of 5-s (each side) Pallof press with a band was performed. For testing, players were in a vertical position, standing straight, and secured above and under their knees with stabilizing pads to prevent leg movement and allow maximal efforts during rotational movement. The height of the trunk modular component was set to individual positions until the dynamometer attachment reached the celiac plexus. Both hands held the Humac NORM wheel attachment at shoulder height while the shoulders were retracted with arms straight (elbow bending during setup and testing was not allowed). Depending on the side of rotation, the hand closer to the dynamometer was always on top (Figure 1). Prior to maximal effort, players were instructed to perform two submaximal 3-s trials at a level between 50% and 70% of their individual MVC with a 10-s rest in between due to familiarization. The testing protocol consisted of four trials of 3 s of rotational full-body pulls with a 30-s rest between trials. The test was performed for the dominant and non-dominant sides. Intra-rater reliability was calculated before data analysis by intra-class correlation coefficient (ICC = 0.979) with the standard error of measurement = 3.07 (%SEM = 13.38%) and the smallest detectable change = 4.86 (SDC% = 21.17%). This reliability refers to the consistency of the data recorded by one rater over several trials and is best determined when multiple trials are administered over a short period of time (Scheel et al., 2018).

#### 2.3.7 Eccentric strength of knee flexors

Eccentric peak force in newton of knee flexors (NB_ECC) was tested on the NordBord device (Vald Performance, Queensland, Australia) in a kneeling position at a sampling frequency of 50 Hz. High test–retest correlations of eccentric peak force (left leg: r = 0.906, CI = 0.798–0.962; right leg: r = 0.792, CI = 0.571–0.924) on NordBord devices in elite football players were reported by Rhodes et al. (2022). Per the protocol described by Drury et al. (2020), players kneeled on the board with their ankles fixed by an individual hook for each leg (uniaxial load cell), hips fully extended, and arms across the chest. Players were instructed to move forward and lower their upper body with the slowest possible speed to achieve their maximal prone position until failure. A single set of three repetitions was performed with 30-s rest between each repetition.

#### 2.3.8 Isometric strength of hip adductors

The isometric bilateral peak force in newton of hip adductors (FF_ADD) was tested on the ForceFrame Strength Testing System device (Vald Performance, Queensland, Australia) at a sampling frequency of 400 Hz. Ryan et al. (2019) reported a high test–retest reliability of GroinBar adductor strength testing using the Vald Performance system in professional Australian footballers (ICC = 0.87–0.96, %CV = 6.3, and %SWC = 5.0). The players were in a lying supine position with knees flexed at 45°. They performed three maximal voluntary isometric contractions (5 s) when they pushed their knees into the sensors (load cells), which were placed on the medial malleoli femoral condyles, followed by a minimum of 10 s of rest between trials (Desmyttre et al., 2019). Excellent reliability and minimal detectable change were reported in previous studies (Desmyttre et al., 2019).

#### 2.3.9 Vertical ground reaction force in jumps

Force differences in power assessment (vertical jump test) were examined by two Kistler 8611 force plates (Kistler Group, Winterthur, Switzerland) at a sampling frequency of 1,000 Hz and using software (BioWare 5.4.3.0, Winterthur, Switzerland). Hori et al. (2009) reported the following coefficient of reliability for the Kistler force plate: peak force: ICC = 0.92, peak velocity: ICC = 0.98, and peak power: ICC = 0.98. The peak vertical ground reaction forces (VGRFs) in newton exerted under each foot separately were examined during the take-off phase of four different jump tasks: counter-movement jump-free arms (CMJFA), counter-movement jump (CMJ), squat jump (SJ), and drop jump (DJ). Based on the height of each jump type, the best of three attempts was selected for data processing.

### 2.4 Data processing

Descriptive statistics were calculated for all variables (mean, standard deviation, 95% confidence interval, and range). The normality of the data distribution was verified using the Shapiro–Wilk test. Research data were processed using a one-way ANOVA followed by multiple comparisons of Bonferroni’s *post hoc* tests. Pearson’s coefficient (r) and coefficient of determination (R^2^) were used to examine the relationships between the investigated variables. Statistical significance was set at *p* < 0.05. Partial eta squared (η_p_^2^) was also calculated, and effect sizes were estimated as follows: between ≥0.01 and <0.06—small, between ≥0.06 and <0.14—medium, and ≥0.14—large (Richardson, 2011). Statistical analysis was carried out using IBM^®^ SPSS^®^ v21 (Statistical Package for Social Science, Inc., Chicago, IL, 2012).

## 3 Results

### 3.1 Magnitude of asymmetries

**FIGURE 2:**
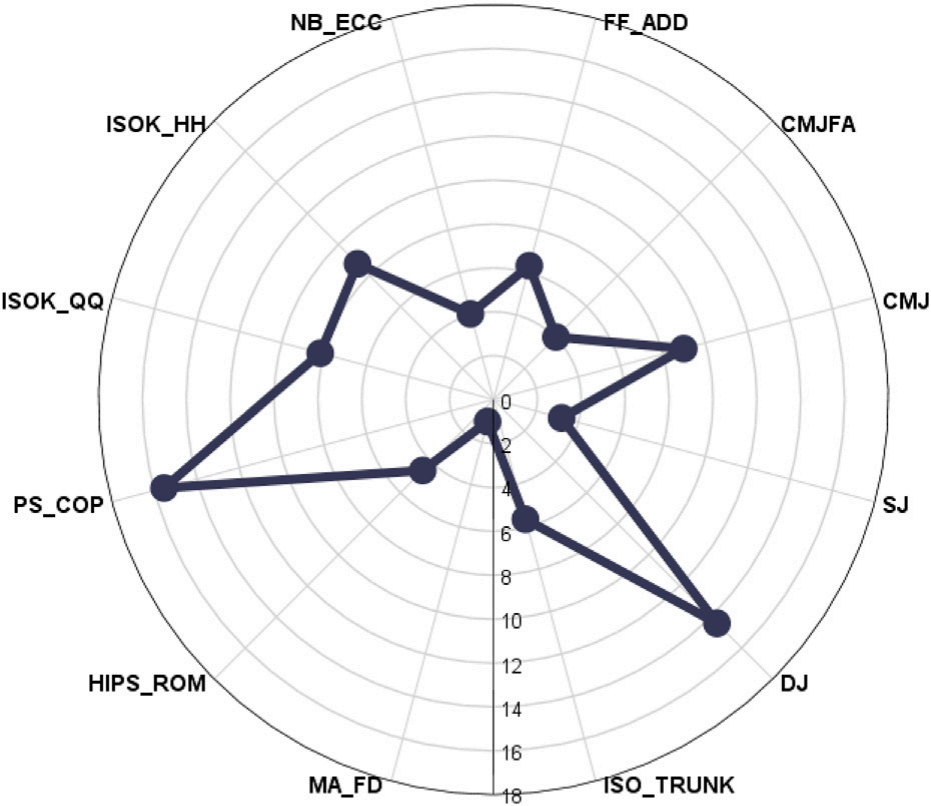
Level of asymmetries in the measured parameters. Data are expressed in relative values (%). MA_FR, morphological asymmetries; HIPS_ROM, range of motion asymmetries; PS_COP, neuromuscular asymmetry; ISOK_QQ, isokinetic strength of knee extensors; ISOK_HH, isokinetic strength of knee flexors; NB_ECC, eccentric strength of knee flexors; FD_ADD, isometric strength of hip adductors; CMJFA, vertical ground reaction force asymmetry in counter-movement jump with arms; CMJ, vertical ground reaction force asymmetry in counter-movement jump; SJ, vertical ground reaction force asymmetry in squat jump; and DJ, vertical ground reaction force asymmetry in the drop jump test.

Significant differences of asymmetries were found in elite soccer players (F_11,299_ = 11.01, *p* < 0.01). While the highest level of asymmetries between the dominant and non-dominant sides were found in PS_COP (15.54% ± 9.76%, CI95 = 11.51–19.57%) and DJ (14.40% ± 13.00%, CI95 = 9.04–19.77%), the lowest asymmetries were in fluid distribution of lower limbs, MA_FD (1.03% ± 1.15%, CI95 = 0.50–1.50%), and in the SJ test, where VGRF difference during the take-off phase was 3.22% ± 0.91% (CI95 = 2.02–4.42%; Figure 2). *Post hoc* analysis revealed significant differences between the variables (Table 2). The magnitudes of measured asymmetries between ISOK_QQ, ISOK_HH, NB_ECC, and FF_ADD were insignificant (*p* > 0.05). The VGRF difference between SJ and DJ was significant (3.22% ± 2.91% vs 14.41% ± 13.00%).

**TABLE 2:** Levels and differences between asymmetries (the letter in *post hoc* means a significant difference with the variable in the same row).

Variable	Mean ± SD	95% CI	Range	*Post hoc*
MA_FD^a^	1.03 ± 1.15	0.56–1.50	3.64	c, d, e, h, k
HIPS_ROM^b^	4.56 ± 3.41	3.15–5.97	11.90	c, k
PS_COP^c^	15.54 ± 9.76	11.51–19.57	37.68	a, b, d, e, f, g, h, i, j, l
ISOK_QQ^d^	8.17 ± 7.24	5.18–11.16	26.60	a, c
ISOK_HH^e^	8.76 ± 6.29	6.16–11.36	22.90	a, c
NB_ECC^f^	4.04 ± 4.89	2.03–6.07	23.88	c, k
FF_ADD^g^	6.32 ± 4.56	4.44–8.20	16.15	c, k
CMJFA^h^	4.03 ± 3.35	2.65–5.41	11.82	c, k
CMJ^i^	8.98 ± 8.83	5.33–12.63	33.20	a, c
SJ^j^	3.22 ± 2.91	2.02–4.42	13.84	c, k
DJ^k^	14.41 ± 13.00	9.04–19.77	46.20	a, b, f, g, h, j, l
ISO_TRUNK^l^	5.64 ± 3.64	4.14–7.14	14.00	c, k

MA_FD, morphological asymmetries; HIPS_ROM, range of motion asymmetries; PS_COP, neuromuscular asymmetry; ISOK_QQ, isokinetic strength of knee extensors; ISOK_HH, isokinetic strength of knee flexors; NB_ECC, eccentric strength of knee flexors; FD_ADD, isometric strength of hip adductors; CMJFA, vertical ground reaction force asymmetry in counter-movement jump with arms; CMJ, vertical ground reaction force asymmetry in counter-movement jump; SJ, vertical ground reaction force asymmetry in squat jump; DJ, vertical ground reaction force asymmetry in the drop jump test; SD, standard deviation; CI, confidence interval.

### 3.2 Correlations between asymmetries

Eleven significant correlations (*p* < 0.05, r = 0.41–0.63, *R* ^2^ = 0.17–0.40; Table 3) were detected between the examined asymmetries in elite soccer players. The highest relationships were revealed between CMJFA vs. CMJ (r = 0.63, *R* ^2^ = 0.40, d = moderate), MA_FD vs. ISOK_HH (r = 0.61, *R* ^2^ = 0.37, d = moderate), CMJ vs. DJ (r = 0.58, *R* ^2^ = 0.34, d = moderate), and ISO_TRUNK vs. DJ (r = 0.56, *R* ^2^ = 0.31, d = moderate). HIPS_ROM and PS_COP showed no relationship with any other parameter (*p* > 0.05).

**TABLE 3:** Relationships between morphological, strength, and neuromuscular asymmetries.

	MA_FD	HIPS_ROM	PS_COP	ISOK_QQ	ISOK_HH	NB_ECC	FF_ADD	CMJFA	CMJ	SJ	DJ	ISO_TRUNK
MA_FD	**1**											
HIPS_ROM	−.11	**1**										
PS_COP	−.18	.34	**1**									
ISOK_QQ	.08	−.17	−.26	**1**								
ISOK_HH	**.61** ^**^	−.26	−.08	−.12	**1**							
NB_ECC	.31	−.24	.04	.02	**.41** ^*^	**1**						
FF_ADD	.29	−.09	−.08	−.32	.08	−.13	**1**					
CMJFA	−.01	−.08	.06	.24	.14	.13	−.11	**1**				
CMJ	−.19	−.10	.15	.16	−.07	−.08	−.21	**.63** ^**^	**1**			
SJ	−.01	.03	.17	.30	.19	.07	**−.48** ^*^	**.45** ^*^	**.51** ^**^	**1**		
DJ	−.01	−.18	.06	**.42** ^*^	−.16	−.18	−.12	**.44** ^*^	**.58** ^**^	.20	**1**	
ISO_TRUNK	.33	−.13	−.10	**.43** ^*^	.00	.09	.11	.36	.28	.33	**.56** ^**^	**1**

MA_FD, morphological asymmetries; HIPS_ROM, range of motion asymmetries; PS_COP, neuromuscular asymmetry; ISOK_QQ, isokinetic strength of knee extensors; ISOK_HH, isokinetic strength of knee flexors; NB_ECC, eccentric strength of knee flexors; FD_ADD, isometric strength of hip adductors; CMJFA, vertical ground reaction force asymmetry in counter-movement jump with arms; CMJ, vertical ground reaction force asymmetry in counter-movement jump; SJ, vertical ground reaction force asymmetry in squat jump; DJ, vertical ground reaction force asymmetry in the drop jump test.

## 4 Discussion

Eleven out of a total of sixty six analyzed relationships were significantly correlated in a group of elite players. Those most strongly related to each other were the jump tests. The counter-movement jump has been analyzed extensively in the literature, with results indicating a typical asymmetry in vertical ground reaction force of 0.9%–7.0% (Menzel et al., 2013; Yanci and Camara, 2016; Nicholson et al., 2022). Asymmetries as low as 5% in CMJ testing have been found to be associated with decreased physical performance during sports tasks (Bishop et al., 2021). Our results found 5.6%–7.1% asymmetries in our two CMJ movements. This indicates that there is still room for elite soccer players to improve this asymmetry. The drop jump has been analyzed previously but without vertical ground reaction force (Arundale et al., 2020). The results presented here show a moderate correlation between all jump tests, except between SJ and DJ. Since these jumps represent different measures of the stretch-shortening cycle, it is interesting to find that the test of the fastest reaction (slowest possible ground time-contact control in drop jump) had the largest SA of the jump tests (14.41% on average). Given that 10%–15% is often used as a cut-off for jump asymmetry assessment (Menzel, et al., 2013), elite players may still require an intervention to limit potential performance deficits and reduce injury risk. Moreover, high-level reactive jump training, with a focus on balanced load (force) production, may be required to reduce the large asymmetry in the DJ.

Lower-limb muscle strength values were below 10% (except VGRF in DJ), which is generally accepted as a “low asymmetry.” This is in line with more recent research (Lutz et al., 2022), as elite male soccer players have benefitted from the intervention programs implemented to reduce asymmetries, as indicated by previous studies (Tyler et al., 2001; Fousekis et al., 2010a). On the other hand, we need to pay attention to individual player strength and asymmetric profile instead of group mean assessment because large inter-individual values were found (Table 2). Constant loading of one side of the body over time (passing, shooting, ball dribbling, and specific movement patterns) may lead to strength asymmetry and imbalances in tissue adaptation. Among the strength asymmetry variables, it is interesting to note that the relationship between the NordBord eccentric hamstring strength and the isokinetic concentric hamstring strength was only moderate (r = 0.407). A similar correlation (r = 0.35) between eccentric strength in “Nordic hamstring curls” (NordBord device) and the isokinetic test in professional soccer players (n = 306) has been reported by van Dyk et al. (2018). This reinforces the point that both concentric and eccentric hamstring strength should be tested in elite soccer players as their asymmetries cannot be interchanged. Another key aspect was that eccentric strength on the NordBord device was tested bilaterally in the closed kinetic chain, while testing on isokinetic dynamometry was performed unilaterally in the open kinetic chain and concentric muscle contraction. Different lower limb muscle asymmetries were associated with different jump tests. Adductor asymmetry was significantly correlated to SJ, which may be because the reduced movement of the hips requires more adductor input into the jumping movement (Yoshioka et al., 2011). The quadriceps asymmetry was significantly correlated to DJ, with this test requiring the most elastic energy activity from the quadriceps (Peng et al., 2011). Connected to both of these variables is the isometric trunk-rotation strength, which was moderately correlated to both asymmetries. This represents an interconnection between the asymmetry of the trunk, quadriceps, and DJ. This indicates a multifaceted approach and adaptations to reactive jumping in elite soccer players (Hammami et al., 2019). This is a novel relationship that has not been identified previously. This result can be used in designing strengthening programs by practitioners, especially when ISO_TRUNK rotation strength testing was just one of the tests that were performed in the transverse plane. Strength testing of players in the transverse plane may be beneficial for players and practitioners as soccer players move their whole-body segments to produce and transfer mechanical energy in all three planes during soccer movements. It has been reported that trunk–pelvic motion in the transverse plane is related to sports performance in soccer (Fonseca et al., 2011), but research has also shown asymmetry in the trunk–pelvic stabilization in the transverse plane in soccer players regardless of limb dominancy or field position (Santos et al., 2014).

Hamstring strength asymmetry (ISOK_HH) was significantly correlated with fluid distribution in lower limbs (morphological asymmetry). This can be explained through biomechanical and physiological considerations. If one lower limb exhibits greater strength repetitively, it may affect muscle activation during movement (Mertz et al., 2019). Fluid asymmetry, often observed as interstitial fluid distribution changes, may be a consequence of altered biomechanics and load repetition (Pereira and Shefelbine, 2014). The muscles play a role in the lymphatic system’s function, which helps regulate fluid balance (Muthuchamy and Zawieja, 2008). Asymmetric muscle forces may impact this system, potentially leading to fluid asymmetry in the affected limb. Moreover, hamstring strength asymmetry can influence joint mechanics and increase the risk of injuries (Fousekis et al., 2010c; Koźlenia et al., 2022; Oleksy et al., 2023), which may further contribute to fluid asymmetry. It is crucial to consider the interconnected nature of the musculoskeletal and fluid systems, recognizing that alterations in muscle strength and function can have cascading effects on fluid dynamics within the body. Additional research is needed to provide a more comprehensive understanding of this link. Postural stability had the largest asymmetry in elite soccer players, a parameter that remains highly asymmetrical (Chaari et al., 2022). Surprisingly, it was not significantly correlated to adductor asymmetry in our results, as previous research has linked groin injury to postural stability asymmetry (Chaari et al., 2022). This may be due to the difference in testing and measures attained between the studies. Like postural stability asymmetry, HIPS_ROM asymmetry was not significantly correlated to any other parameter, even though it is important for kicking a ball (Barbieri et al., 2015). These results indicate that other aspects also play a role instead of just the muscle strength and force generation of the lower limbs, such as flexibility in ROM and visuospatial systems in balance performance (Ocarino et al., 2021; Zemková and Zapletalová, 2022). Studies suggest that integrating the action/motor preference framework can enhance the precision of tailored training programs in soccer by analyzing top players’ motor skills (Castañer et al., 2017), improving various aspects of performance (Slimani et al. 2016; Lee et al., 2020), and helping coaches monitor young players’ progress (Raiola and Altavilla, 2020). Examining players’ motor preferences and their alignment with hemispheric brain dominance, as suggested by Serrien and Sovijärvi-Spapé (2015), may provide an understanding of the observed asymmetries. Integrating the action/motor preference framework into the analysis could enhance the precision of tailored training programs, considering individual variations in motor dominance within the context of soccer performance.

While this research highlights significant correlations between lower limb and trunk strength in elite soccer players, it is crucial to recognize that these associations do not inherently imply a causal relationship. Factors such as training methodologies, individual biomechanics, and other confounding variables may contribute to the observed correlations. Another limitation is that the results cannot be generalized to amateur, youth, or female players, all of whom present with even higher percentages of asymmetries (Arundale et al., 2020; Maly et al., 2021; Bishop et al., 2022b), as we focused only on male elite soccer players. Future research will benefit from a larger sample size (n > 25) to generalize the conclusions to a broader population of elite soccer players. Additionally, future research may consider, where possible, other contextual factors, such as participants’ injury history, which could also influence the strength or morphological asymmetries. The influence of lower-limb asymmetry linked to trunk strength on high-level soccer training may vary based on the specific positions of the players. Different player positions demand distinct physical attributes, movement patterns, and performance requirements (Arjol-Serrano et al., 2021). We recommend analyzing player position differences in future research, as it may reveal more detailed information to tailor training interventions. In future analysis, we encourage carefully considering these complexities, acknowledging that establishing causation requires further investigation through controlled experiments or longitudinal studies. Maintaining a functional ‘baseline’ asymmetry and its impacts should be the focus of future research, as the statement highlights the complexity of the relationship between reducing asymmetry below a certain threshold, such as 10%, and a decrease in injury risk. Objective evaluations must be used to identify any abnormalities, as it is recognized that some degree of asymmetry may be inherent and natural for soccer players, while an increasing number may exhibit abnormalities. Reducing something may not necessarily result in injury prevention, as current scientific evidence is not in agreement with the baselines. However, the majority of research on elite soccer players suggests that strength asymmetries can impact performance, particularly in short sprints and dynamic tasks, and increase the risk of injury, especially hamstring strains and muscular imbalances (Lehance et al., 2008; Fousekis et al., 2010a; Sannicandro et al., 2011; Bishop et al., 2021; Ascenzi et al., 2022; Stella et al., 2022).

## 5 Conclusion

The magnitude of asymmetries in elite soccer players varied by each parameter, from lower than 5% (MA_FD, HIPS_ROM, NB_ECC, CMJFA, SJ), asymmetries from 5% to 10% (ISOK_QQ, ISOK_HH, FF_ADD, CMJ, ISO_TRUNK), and those over 10% (PS_COP, DJ). The players with long lengths of exposure to soccer practice may develop asymmetry in different body segments. Morphological asymmetries were linked with knee flexor asymmetries (ISOK_HH). The novelty of this study was that lateral trunk-rotation asymmetries (ISO_TRUNK) were significantly correlated to VGRF asymmetries in power assessment (DJ) and knee extensors in elite soccer players. The results indicate that unilateral lower-limb load and its power characteristics at the elite soccer level may influence the development of abnormal strength and morphological adaptations in favor of the dominant side and also in the strength parameters of the trunk. Higher attention should be paid to players who had higher asymmetries (>10%) and those who suffer from hamstring and adductors muscle group based on individual assessment. Clinicians and conditioning practitioners should be aware of monitoring whole-body strength throughout the season and intentionally intervene in abnormalities.

## Data Availability

The raw data supporting the conclusion of this article will be made available by the authors, without undue reservation.
